# Preliminary Study on Insecticidal Potential and Chemical Composition of Five Rutaceae Essential Oils against *Thrips flavus* (Thysanoptera: Thripidae)

**DOI:** 10.3390/molecules28072998

**Published:** 2023-03-28

**Authors:** Tian-Hao Pei, Yi-Jin Zhao, Sheng-Yuan Wang, Xiao-Feng Li, Chen-Qi Sun, Shu-Sen Shi, Meng-Lei Xu, Yu Gao

**Affiliations:** 1 College of Plant Protection/Key Laboratory of Soybean Disease and Pest Control (Ministry of Agriculture and Rural Affairs), Jilin Agricultural University, Changchun 130118, China; 2 Dalian Customs District, Dalian 116001, China; 3 College of Food Science and Engineering, Jilin University, Changchun 130062, China

**Keywords:** essential oil, *Thrips flavus*, insecticidal activity, gastric toxicity, thrips

## Abstract

To meet the demand for novel pest management strategies to combat the development of insecticide resistance, plant essential oils may be a promising alternative source. This study investigated the insecticidal activity of five essential oils from the Rutaceae plant family against *Thrips flavus* Schrank (Thysanoptera: Thripidae) under laboratory conditions. The plant essential oils were citrus oil (*Citrus reticulata* Blanco), Chuan-shan pepper oil (*Zanthoxylum piasezkii* Maxim.), zanthoxylum oil (*Zanthoxylum bungeanum* Maxim.), pomelo peel oil (*Citrus maxima* (Burm.) Merr.) and orange leaf oil (*Citrus sinensis* (L.) Osbeck). Among the essential oils evaluated, orange leaf oil (LC_50_ = 0.26 g/L), zanthoxylum oil (LC_50_ = 0.27 g/L), and pomelo peel oil (LC_50_ = 0.44 g/L) resulted in a higher gastric toxicity under laboratory conditions. The results of the pot experiment also showed that orange leaf oil (93.06 ± 3.67% at 540.00 g a.i.·hm^−2^, 97.22 ± 1.39% at 720 g a.i.·hm^−2^, 100.00% at 900.00 g a.i.·hm^−2^) zanthoxylum oil (98.73 ± 1.27% at 900 g a.i.·hm^−2^), and pomelo peel oil (100.00% at 900 g a.i.·hm^−2^) exhibited a higher control efficacy, being the most effective against *T. flavus* after 7 days of treatment. The essential oil components were then identified by gas chromatography–mass spectrometry (GC–MS). The insecticidal activity of orange leaf oil, pomelo peel oil, and zanthoxylum oil could be attributed to their main constituents, such as methyl jasmonate (50.92%), D-limonene (76.96%), and linalool (52.32%), respectively. In the olfactory test, adult *T. flavus* were attracted by zanthoxylum oil and Chuan-shan pepper oil. We speculated that linalool might be the key signaling compound that attracts *T. flavus*. These results showed that orange leaf oil, zanthoxylum oil, and pomelo peel oil exhibited insecticidal activities under controlled conditions. They can be implemented as effective and low-toxicity botanical insecticides and synergistic agents against *T. flavus*.

## 1. Introduction

Eurasian yellow flower thrips, *Thrips flavus* Schrank (Thysanoptera: Thripidae), is a worldwide phytophagous pest distributed in Asia and Europe and recorded from a wide range of host plants [1,2,3]. *T. flavus* is a host to a strain of the tomato spotted wilt virus (TSWV-W) on watermelon (*Citrullus lanatus* (Thunb.) Matsum. *et* Nakai) in India [4,5], which is highly invasive to other regions since it has well-developed dispersion mechanisms [6]. *T. flavus* has been increasingly recognized as a dominant pest of Compositae (Asteraceae), Leguminosae (Fabaceae), and other plant families during the flowering stage in north China [7]. This pest generally results in curling, deformation, and withering of the leaves and early senescence or deformation of the flowers [8]. Currently, the field management of *T. flavus* depends on chemical insecticides [9,10]. However, long-term insecticide use would lead to the rapid development of insecticide resistance in pest thrips [11,12]. An effective way to reduce resistance incidence is to use botanical insecticides instead of chemical insecticides [13]. Plant-derived essential oil products originating from various insecticidal plant species are of great concern for alternative products expected to solve insect resistance development [13,14]. Plant essential oils are natural volatiles and fragrant compounds produced as bioactive secondary metabolites [15]. Essential oils (EOs) can be applied against pests in two different ways: by spraying crops or by fumigating plants grown in greenhouses [16]. EOs can also be used as insect antifeedants [17], repellents [18], growth regulators, and reproduction inhibitors [19]. Previous studies have been conducted to assess the insecticidal effects and cytotoxicity of plant EOs on different pests. EOs from *Cynanchum mongolicum* (Maxim.) Kom. (Asclepiadaceae) have insecticidal activities against soybean aphid, *Aphis glycines* Matsumura (Hemiptera: Aphidiae) [20]. A combination of *Acorus tatarinowii* Schott (Araceae) and *Stemona japonica* (Blume) Miq. (Stemonaceae) essential oils were also effective against *A. glycine* [21]. EOs from *Croton grewioides* Baill (Euphorbiaceae) and *Piper aduncum* L. (Piperaceae) were both effective against soybean looper, *Chrysodeixis includens* (Walker) (Lepidoptera: Noctuidae) [22,23]. EOs from *Aloysia polystachya* (Griseb.) Moldenke, *Aloysia citriodora* Ortega ex Pers. (Verbenaceae), *Origanum vulgare* L., and *Thymus vulgaris* L. (Labiatae) were effective against the Southern green stink bug, *Nezara viridula* (L.) (Hemiptera: Pentatomidae) [24,25]. EOs from *Piper aduncum* L. (Piperaceae) can cause fat body cellular stress in the brown stink bug, *Euschistus heros* (F.) (Heteroptera: Pentatomidae) [26], and *Anticarsia gemmatalis* Hübner (Lepidoptera: Noctuidae) [27].

Among the numerous essential oil-bearing plants, the Rutaceae family plants (order Sapindales) are widely distributed worldwide in tropical and temperate regions. They are mainly used for fresh consumption and traditional medicine [28]. The leaf, peel, and flesh can also have potential medicinal and agrochemical applications [29]. If these agricultural by-products are not properly disposed of, the waste would seriously burden the environment. Thus, their overall value can be greatly enhanced if they are fully utilized [30,31]. One of the effective ways to fully develop and utilize these resources is to study the bioactive components of essential oils from the Rutaceae family [32]. Rutaceae essential oils contain alkaloids, flavonoids, terpenoids, sesquiterpene, steroids, and other compounds, in a wide range of concentrations [29,33]. The search for compounds with insecticidal activity facilitates the development of innovative pesticides and the utilization of postharvest agricultural by-products that would otherwise be wasted in most cases [34,35]. These bioactive components have been reported as having insecticidal potential against a wide range of insect pests, such as *Aedes albopictus* (Skuse) (Diptera: Culicidae) [36], *Culex quinquefasciatus* (Say) (Diptera: Culicidae) [37], *Bemisia tabaci* (Gennadius) (Hemiptera: Aleyrodidae) [38], *Tribolium castaneum* Herbst (Coleoptera: Tenebrionidae) [39], *Spodoptera frugiperda* (J. E. Smith) (Lepidoptera: Noctuidae) [40], *Agrotis ipsilon* (Lepidoptera: Noctuidae) [41], *Callosobruchus maculatus* L., *Sitophilus zeamais* Motschulsky (Coleoptera: Curculionidae) [42,43], etc. However, the insecticidal effect of Rutaceae essential oil on *T. flavus* is still unknown. The present work aimed to investigate the insecticidal activities in *T. flavus* of five essential oils from the Rutaceae plant family under laboratory conditions and preliminarily identify the chemical composition of the most bioactive compounds using a gas chromatography–mass spectrometry (GC–MS) analysis. The results will be exploited to develop new botanical insecticides based on potentially bioactive compounds useful for the protection of cultivated crops against *T. flavus*.

## 2. Results

### 2.1. Chemical Analysis of Essential Oils

#### 2.1.1. Chemical Constituents of Citrus Oil

A total of four chemical compounds were identified in citrus oil (Table 1). The most abundant compounds were D-limonene (95.62%), followed by beta-myrcene (2.91%), alpha-pinene (0.90%), and sabenene (0.57%).

#### 2.1.2. Chemical Constituents of Chuan-Shan Pepper Oil

A total of 10 chemical compounds were identified in pepper oil (Table 1). The most abundant compounds in Chuan-shan pepper oil were linalool (64.10%), followed by D-limonene (11.83%) and sabenene (8.29%).

#### 2.1.3. Chemical Constituents of Zanthoxylum Oil

A total of six chemical compounds were identified in zanthoxylum oil (Table 1). The most abundant compounds were linalool (52.69%), followed by D-limonene (22.15%), sabenene (17.13%), and beta-myrcene (3.70%).

#### 2.1.4. Chemical Constituents of Pomelo Peel Oil

A total of 12 chemical compounds were identified in pomelo peel oil (Table 1). The most abundant compounds were D-limonene (78.36%), followed by linalyl anthranilate (5.47%), butylated hydroxytoluene (5.34%), and beta-myrcene (2.81%).

#### 2.1.5. Chemical Constituents of Orange Leaf Oil

A total of 12 chemical compounds were identified in orange leaf oil (Table 1). The most abundant compounds were methyl jasmonate (51.50%), methyl dihydrojasmonate (14.48%), alpha-hexyl cinnamaldehyde (13.33%), and linalool (8.02%).

### 2.2. Laboratory Bioassay

In the gastric toxicity test, the orange leaf oil had the highest biological activity, with an LC_50_ of 0.26 g/L, followed by zanthoxylum oil, with an LC_50_ of 0.27 g/L, and pomelo peel oil, with an LC_50_ of 0.44 g/L. Citrus oil was the least active, with an LC_50_ of 2.73 g/L (Table 2). This indicated that among the Rutaceae EOs tested, the orange leaf oil, zanthoxylum oil, and pomelo peel oil possessed a higher gastric toxicity.

### 2.3. Pot Experiments

#### 2.3.1. Control Efficacy of Citrus Oil against *T. flavus*

The control efficacy of citrus oil against *T. flavus* increased with increasing concentration and treatment time (Table 3). Among them, except for the concentrations of 900.00 and 3600.00 g a.i.·hm^−2^, from day 1 to day 7, the control efficacy initially showed an increasing trend, which then decreased. Notably, the differences between the concentrations evaluated diminished as the treatment time increased from 1 day to 7 days (Table 3). The control efficacy of 900 g a.i.·hm^−2^ was significantly lower than that of 2700.00, 3600.00, and 4500.00 g a.i.·hm^−2^ after 1 day (*F* = 9.3950, *df* = 4, *p* = 0.002). No significant differences were observed between the five concentrations after 3 days (*F* = 0.3070, *df* = 4, *p* = 0.8666) and after 7 days (*F* = 0.3960, *df* = 4, *p* = 0.8069). The highest control efficacy of citrus oil was 62.03 ± 4.38% at 4500.00 g a.i.·hm^−2^ after 7 days.

#### 2.3.2. Control Efficacy of Chuan-Shan Pepper Oil against *T. flavus*

The control efficacy of Chuan-shan pepper oil against *T. flavus* increased with the increasing application concentration and treatment time (Table 3). The control efficacy of 540.00 g a.i.·hm^−2^ was significantly higher than that of 180.00 g a.i.·hm^−2^ after 1 day (*F* = 3.9900, *df* = 4, *p* = 0.0346). The control efficacy of 900.00 g a.i.·hm^−2^ was significantly higher than that of 180.00 and 360.00 g a.i.·hm^−2^ after 3 days (*F* = 23.2200, *df* = 4, *p* = 0.0001). The highest control efficacy of Chuan-shan pepper oil was 82.28 ± 3.35% at 900.00 g a.i.·hm^−2^ after 7 days (*F* = 24.9680, *df* = 4, *p* = 0.0001).

#### 2.3.3. Control Efficacy of Zanthoxylum Oil against *T. flavus*

The control efficacy of zanthoxylum oil against *T. flavus* similarly increased with the increasing concentration and treatment time (Table 3). The control efficacy of 900.00 g a.i.·hm^−2^ was significantly higher than that of 180.00, 360.00, and 540.00 g a.i.·hm^−2^ after 1 day (*F* = 12.5140, *df* = 4, *p* = 0.0007). The control efficacy of 720.00 and 900.00 g a.i.·hm^−2^ was significantly higher than the other applied concentrations after 3 days (*F* = 11.7720, *df* = 4, *p* = 0.0008). The highest control efficacy of zanthoxylum oil was 98.73 ± 1.27% at 900.00 g a.i.·hm^−2^ after 7 days (*F* = 37.106, *df* = 4, *p* = 0.0001).

#### 2.3.4. Control Efficacy of Pomelo Peel Oil against *T. flavus*

The control efficacy of pomelo peel oil against *T. flavus* increased with the increasing concentration and treatment time (Table 3). The control efficacy of 900.00 g a.i.·hm^−2^ was the highest compared to the other applied concentrations after 1 day (*F* = 26.1330, *df* = 4, *p* = 0.0001). The control efficacy of 900.00 g a.i.·hm^−2^ was significantly higher than that of 180.00 and 360.00 g a.i.·hm^−2^ after 3 days (*F* = 17.8250, *df* = 4, *p* = 0.0002) and reached its highest value after 7 days with 100.00% efficacy (*F* = 37.2250, *df* = 4, *p* = 0.0001), which was the highest control efficacy of pomelo peel oil measured overall.

#### 2.3.5. Control Efficacy of Orange Leaf Oil against *T. flavus*

The control efficacy of orange leaf oil against *T. flavus* increased with the increasing concentration and treatment time (Table 3). The control efficacy of 720.00 and 900.00 g a.i.·hm^−2^ was significantly higher than the other concentrations after 1 day (*F* = 11.601, *df* = 4, *p* = 0.0009). The control efficacy of 900.00 g a.i.·hm^−2^ was significantly higher than the other concentrations evaluated after 3 days (*F* = 18.9550, *df* = 4, *p* = 0.0001). The control efficacy of 720.00 and 900.00 g a.i.·hm^−2^ was significantly higher than that of 180.00 and 360.00 g a.i.·hm^−2^ after 7 days (*F* = 11.4030, *df* = 4, *p* = 0.0001). The highest control efficacy of orange leaf oil was 100.00% at 900.00 g a.i.·hm^−2^ after 7 days.

Of the five essential oils tested, those with a control efficacy above 90% after 7 days were the orange leaf oil, with 93.06 ± 3.67% at 540.00 g a.i.·hm^−2^, 97.22 ± 1.39% at 720 g a.i.·hm^−2^, and 100.00% at 900.00 g a.i.·hm^−2^; the pomelo peel oil with 100.00% at 900 g a.i.·hm^−2^; and the zanthoxylum oil with 98.73 ± 1.27% at 900 g a.i.·hm^−2^.

### 2.4. Olfactometer Test

**Figure 1:**
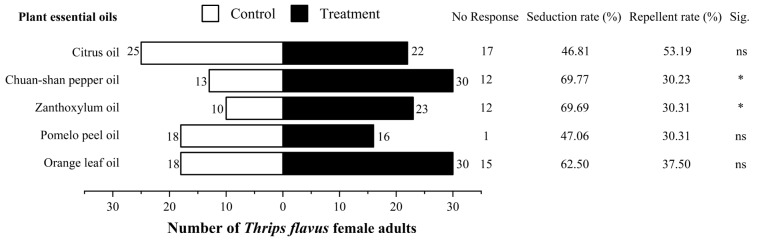
Olfactory behavioral response of *Thrips flavus* to five Rutaceae essential oils.

*T. flavus* was significantly attracted to Chuan-shan pepper oil and zanthoxylum oil (*χ^2^_Chuan-shan pepper oil_* = 3.888, *P_Chuan-shan pepper oil_* = 0.049; *χ^2^_zanthoxylum oil_* = 4.767, *P_zanthoxylum oil_* = 0.029), and the seduction rate was 69.77% and 69.69%, respectively. The other essential oils did not result in a significant olfactory-induced attraction of *T. flavus* (Figure 1).

## 3. Discussion

In agricultural pest control, pesticide resistance is becoming increasingly prominent; thus, a higher demand exists for developing and applying efficient and safe insecticides [5]. Plant essential oils can be used in pest thrips management strategies and reduce the overuse of synthetic pesticides [44]. The present study was conducted to evaluate the insecticidal activity of five Rutaceae EOs for *T. flavus* control, followed by a preliminary identification of their chemical composition. Among the EOs tested, orange leaf oil (LC_50_ = 0.26 g/L), zanthoxylum oil (LC_50_ = 0.27 g/L), and pomelo peel oil (LC_50_ = 0.44 g/L) showed the highest gastric toxicity. The pot experiments’ results demonstrated that orange leaf oil (93.06 ± 3.67% at 540.00 g a.i.·hm^−2^, 97.22 ± 1.39% at 720 g a.i.·hm^−2^, 100.00% at 900.00 g a.i.·hm^−2^), zanthoxylum oil (98.73 ± 1.27% at 900 g a.i.·hm^−2^), and pomelo peel oil (100.00% at 900 g a.i.·hm^−2^) had the highest control efficacy and were the most effective against *T. flavus* after 7 days of treatment. EOs are complex mixtures of secondary plant metabolites with a significant chemical variability [45,46]. Therefore, the individual or combined properties of the Rutaceae EOs tested against *T. flavus* could potentially be used to develop alternative pesticides for crop protection.

As we emphasized the control efficacy of the EOs against *T. flavus*, only a preliminary chemical analysis of the EOs was performed (Figure S1). The chemical composition of the tested EOs was, in part, consistent with previous reports [30,47,48,49]. Limonene is the major constituent of EOs extracted from citrus by-products, whereas linalool, myrcene, sabinene, and pinene are characteristic of the Rutaceae EOs [48,49,50]. However, certain EOs’ constituents differed from those reported in other studies. For example, the analysis of essential oil from the orange peel parts of *C. sinensis* from Argentina identified limonene (90.48%) as the most prominent compound [49]. The main constituents of *Zanthoxylum bungeanum* essential oil, extracted using a low-eutectic solvent and steam distillation, were terpineol-4-ol (13.13%) and (–)-β-pinene (11.17%) [51]. Many studies have shown that differences in the prominent essential oil compounds could be explained by variables such as harvest time, plant ontogeny, geographical location, plant parts, as well as extraction techniques [52,53,54,55,56].

Therefore, we suggest that the insecticidal activity of pomelo peel oil, orange leaf oil, and zanthoxylum oil may be attributed to their main constituents, such as D-limonene (78.36%), methyl jasmonate (51.50%), and linalool (52.69%), individually, or synergistically to the other active compounds that are present in lower concentrations [16]. Previous studies have also shown that these Rutaceae EO compounds are known for their insecticidal activity against many insects [57,58,59,60,61]. For example, the main compound of *Protium heptaphyllum* (Aubl.) (Sapindales: Burseraceae), D-limonene, exhibited contact and fumigation toxicity and had an ovicidal activity on *C. maculatus* (Coleoptera: Chrysomelidae: Bruchinae) [57]. D-limonene from celery (*Apium graveolens* L.) seeds and roots exhibited contact toxicity against *T. castaneum*, *Lasioderma serricorne* (Coleoptera: Anobiidae), and *Liposcelis bostrychophila* (Psocoptera: Liposcelididae) with LD_50_ values of 14.57 μg/adult, 13.91 μg/adult, and 810.85 μg/cm^2^ [58]. D-limonene exhibited contact toxicity (ranging from 132.48 to 828.79 μg/cm^2^) and fumigant toxicity (LC_50_ = 4.55 mg/L, 7.92 mg/L) against *S. zeamais* and *Sitophilus oryzae* L. [59]. Linalool and D-limonene exhibited excellent fumigant toxicity against red imported fire ants (*Solenopsis invicta* Buren). Linalool resulted in a 100% mortality rate against red imported fire ant workers at 5, 10, and 20 mg/tube after 8 h of treatment, and D-limonene induced >86% mortality after 8 h of exposure [60]. Linalool demonstrated both contact and fumigant activities against *L. serricorne* (LD_50_ = 15.36 μg/larva) [61]. In addition, the insecticidal activity of the EOs tested depends on the plants they derive from, their concentration, and exposure time [16]. Two common compounds of citrus oil and pomelo peel oil were D-limonene and beta-myrcene, but the control efficacy of the two EOs against *T. flavus* differed considerably. This suggests that alpha-pinene (0.89%), sabenene (0.56%), or other compounds may antagonistically affect essential oil toxicity. One noteworthy observation was the lower activity of the citrus oil in samples with a higher relative percentage. The toxic effect of the EOs was due to the combined activities of different components, either with or without significant individual component toxicity against the target pests, e.g., the EO constituents of *Ocimum kilimandscharicum* and *Ocimum kenyense* (Labiateae) on *S. zeamais* and *Rhyzopertha dominica* and the EO of Piperaceae plants on *Callosobruchus chinensis* (L.) [62,63].

Both zanthoxylum oil and Chuan-shan pepper oil were also attractive to *T. flavus* in the olfactometer test. The GC–MS analysis results revealed linalool as the main component of the two EOs, which may be the key signaling compound that attracts *T. flavus*. Linalool is a common volatile organic compound that mediates host selection, foraging behavior, and other insect life activities. The attractiveness of linalool to thrips or other insects is supported by previous studies reporting the attraction of *Thrips major* Uzel to *Sambucus nigra* L. (Caprifoliaceae) [64], *Thrips obscuratus* (Crawford) to *Lonicera japonica* (Thunberg) (Caprifoliaceae) [65], and of *F. occidentalis* to synthetic linalool [66,67]. As a secondary component of the two EOs, D-limonene was repellent to *Bemisia tabaci* (Gennadius) and *Tetranychus urticae* Koch, whereas sabenene exposure resulted in no significant responses [68,69]. Beta-myrcene, on the other hand, was attractive to both males and females of *Myllocerinus aurolineatus* (Voss) (Coleoptera: Curculionidae) [70].

Developing a botanical pesticide is a lengthy process, starting with identifying plant sources with effective biocide activity and then identifying and characterizing its active ingredients [30,44]. This study focused on the insecticidal activity of five Rutaceae EOs against the target pest *T. flavus* and exploited their potential for a practical field application. The chemical composition of these EOs needs to be further evaluated by more advanced analytical chemistry techniques. Although the current insecticidal efficacy of the EOs is slower than that of faster-acting chemical insecticides, and the exact mechanism of action of the main chemical compounds is still not yet fully understood, they still have a good potential for development and applications [71,72]. As a single crop protection measure, spraying plant EOs may not be sufficiently effective [73,74]. However, their potential to control thysanopteran pests should be fully exploited in integrated pest-management strategies [16].

## 4. Materials and Methods

### 4.1. Insects

Adults of *T. flavus* were collected from soybean fields in the Changchun Jingyue Economic Development Zone of Jilin Province, China (43°48′10″ N, 125°24′38″ E). The thrips were continuously reared on soybean trifoliate leaves in an illuminating incubator (GXZ–380B, Ningbo Jiangnan Instrument Factory, Ningbo, China) under a temperature of 25 ± 1 °C, 70% ± 5% RH, and a 16 h:8 h (L:D) photoperiod [67]. The rearing apparatus was the same as in Gao et al. (2021) [7]. Soybean plants were sown in plastic containers (5 cm × 8 cm diameter, 12 cm height), and then the seedlings were watered 3–5 times per week [75].

### 4.2. Plant Essential Oils

The five plant essential oils used were citrus oil (*Citrus reticulata* Blanco), Chuan-shan pepper oil (*Zanthoxylum piasezkii* Maxim.), zanthoxylum oil (*Zanthoxylum bungeanum* Maxim.), pomelo peel oil (*Citrus maxima* (Burm.) Merr.), and orange leaf oil (*Citrus sinensis* (L.) Osbeck). These EOs were purchased from Ji’an Zhongxiang Natural Plants Co., Ltd., produced at the production base in Jiangxi Province of China. The EOs were extracted by steam distillation.

### 4.3. GC–MS Analysis

Chemical composition analyses of the five tested bioactive EOs were carried out with coupled gas chromatography–mass spectrometry (GC–MS) (QP2010 plus, Shimadzu, Japan) equipped with a DB–5 capillary column (30 m × 0.25 mm i.d., 0.25 µm film, J&W Scientific, Folsom, CA, USA). The liquid samples (1 µL) were dissolved in 98 µL *n*-hexane (>99%, Shanghai Aladdin Biochemical Technology Co., Ltd., Shanghai, China).

The injection port was operated in splitless mode with a constant helium flow of 1 mL·min^−1^. The injector temperature was 230 °C, the ionization potential was 70 eV, and the scan frequency was 2 sec^−1^. Following injection, the column temperature was maintained at 60 °C for 1 min, ramped at 11 °C·min^−1^ to 90 °C, held for 2 min, ramped at 2 °C·min^−1^ to 95 °C, held for 1 min, ramped at 18 °C·min^−1^ to 180 °C, held for 2 min, ramped at 2 °C·min^−1^ to 185 °C, held for 1 min, ramped at 13 °C·min^−1^ to 225 °C, held for 2 min, ramped at 2 °C·min^−1^ to 270 °C, and held for 1 min. The limit of detection (LOD) was approximately 2.0 ng ± 0.4 ng. The compound identification was conducted using the GC–MS solution software application, including the mass spectra library NIST 147 and NIST 27 (National Technical Information Services).

### 4.4. Laboratory Bioassay

The gastric toxicity was tested using the leaf-dipping method [76]. Serial dilutions of the five EOs (0.2, 0.4, 0.6, 0.8, 1.0 g/L) were prepared in acetone for evaluation. Fresh soybean leaves of uniform size, undamaged and free from pests and diseases, were selected, washed with water, and dried naturally. The leaves were immersed for 10 s in the tested EO solution and were subsequently removed. After natural drying, the leaves were placed in a centrifuge tube (50 mL, 30 mm diameter). Thirty adult thrips were added to the tube. The tubes were then quickly sealed with parafilm. Approximately 70 micropores were made in the parafilm, using as much force as possible with an insect pin (2#, 0.38 mm diameter), and the holes were evenly distributed. Thirty thrips were tested for each treatment concentration at three independent replicates. An acetone treatment was used as a control. All treated individuals were kept in incubators at 25 ± 1 °C and 70% ± 5%. Mortality was determined after 24 h. Thrips were considered dead if they did not react when touched with a writing brush [75].

According to the bioassay data, adjusted mortality was calculated as follows [76]:

(1) $$MR(\%)=\frac{ND}{NA}\times 100$$

(2) $$AM(\%)=\frac{MRT-MRC}{1-MRC}\times 100$$

where *MR* is the mortality rate, *ND* is the number of dead insects, *NA* is the number of insects treated, *AM* is the adjusted mortality, *MRT* is the mortality rate of the treatment group, and *MRC* is the mortality rate of the control group.

### 4.5. Pot Experiments

Pot experiments were carried out in a greenhouse (25 ± 2 °C and natural lighting) to determine the effect of the five EOs on *T. flavus* control [7]. Soybeans were planted in batches in pots. A disease- and pest-free soybean seedling was selected and maintained in each pot during the second compound leaf development stage. The pot was then covered with a 200-mesh nylon netting. Based on the results of the laboratory bioactivity tests, serial dilutions of citrus oil and orange leaf oil (900.0, 1800.0, 2700.0, 3600.0, and 4500.0 g a.i.·hm^−2^), as well as pomelo peel oil, zanthoxylum oil, and Chuan-shan pepper oil (180.0, 360.0, 540.00, 720.0, and 900.0 g a.i.·hm^−2^) were prepared in acetone for evaluation, respectively. Each concentration was replicated 3 times, with the treatment without the EOs serving as the control. After spraying 5 mL evenly per pot with a spray bottle, the plants were allowed to dry naturally in a windless place. Thirty adult thrips were introduced per pot. The number of dead individuals was observed and recorded 1 day, 3 days, and 7 days after spraying.

According to experimental data, the control efficacy in pot experiments was calculated as follows:

(3) $$CE=\left(1-\frac{AT\times BC}{BT\times AC}\right)\times 100$$

where *CE* is the control efficacy; AT is the number of insects in the treatment group after treatment with EOs; *BC* is the number of insects in the control group before treatment with EOs; *BT* is the number of insects in the treatment group before treatment with EOs; and *AC* is the number of insects in the control group after treatment with EOs.

### 4.6. Olfactory Test

The attractant/repellent activity of each essential oil to individual *T. flavus* adults was measured using a Y-tube olfactometer, as described by Zhang et al. [77]. The essential oil was tested with a 1 μL volume dropped on filter paper in the odor chamber. Pure humidified air was used as a control. Fluorescent light was set in parallel above the Y-tube to avoid light interference. The average intensity of illumination above the olfactometer was 7800–8000 lux. Both arms of the tube were filled with pure humidified air at a rate of 300 mL/min. A single *T. flavus* adult was placed at the entrance of the olfactometer, and after 5 min, its position in the tube was recorded [78]. The response criteria of *T. flavus* adults were determined as follows: if the adults climbed to more than half the length into one of the tubes and remained for 1 min or more, it was deemed that the adults chose this odor source; if the adults kept still or made no choice after 5 min, it was deemed as a no choice. At least thirty adults were exposed to each essential oil treatment. After the evaluation of five adults, the olfactometer tube was cleaned with acetone (99.5%, Xintong Fine Chemical Co., Ltd., Tianjin, China) and then heated at 100 °C for 30 min. All bioassays were conducted between 9:00 and 15:00 h in a room whose temperature was maintained at 24–27 °C.

### 4.7. Statistical Analysis

The data analysis was carried out using Data Processing System (DPS) software version 13.5, http://www.dpsw.cn/dps_eng/index.html (accessed on 5 March 2023) [78]. A one-way ANOVA analysis was used to evaluate the significance of the differences in adjusted mortality between different EOs applied. Tukey’s HSD test was used at a 5% significance after an arcsine transformation of the adjusted mortality data. The analysis of the median lethal concentration (LC_50_) resulting from the treatment with EOs was determined by a log-probit analysis with 95% fiducial limits. Differences between the number of *T. flavus* adults entering each arm of the olfactometer for each paired treatment were analyzed using the *χ^2^* test. All figures were prepared using GraphPad Prism 8.0 software (GraphPad Software, Boston, MA, USA).

## 5. Conclusions

The results demonstrated that under controlled conditions, orange leaf oil, zanthoxylum oil, and pomelo peel oil had strong insecticidal activities. Thus, they have the potential to be utilized as effective and low-toxicity botanical insecticides and synergistic agents against *T. flavus*. Further studies are needed to develop formulations based on the bioactive molecules of these EOs to be used as novel, more effective, and sustainable biopesticides for *T. flavus* control.

## Figures and Tables

**Table 1:** Chemical constituents of five Rutaceae essential oils.

No.	Compounds ^1^	Relative Percentage (%)				
		Citrus Oil (*Citrus reticulata* Blanco)	Chuan-Shan Pepper Oil (*Zanthoxylum piasezkii* Maxim.)	Zanthoxylum Oil (*Zanthoxylum bungeanum* Maxim.)	Pomelo Peel Oil (*Citrus maxim*a (Burm.) Merr.)	Orange Leaf Oil (*Citrus sinensis* (L.) Osbeck)
1	Alpha-pinene	0.899		0.772	1.100	
2	Sabenene	0.573	8.291	17.131	0.507	
3	(1*S*)-(1)-Beta-pinene			0.336	1.421	
4	Beta-myrcene	2.912	1.874	3.698	2.811	
5	1,1′-Oxydi-2-propanol					0.853
6	2,2′-Oxydipropanol					0.750
7	p-Cymene				0.289	
8	2-(2-Hydroxypropoxy)-1-propanol					1.045
9	d-limonene	95.617	11.832	22.145	78.236	
10	Gamma-terpinene				1.853	
11	Linalool		64.101	52.690		8.018
12	Styralyl acetate				1.760	
13	Alpha-terpineol				0.545	
14	Linalyl anthranilate		1.073	1.145	5.468	0.441
15	(–)-Isocaryophyllene			0.658		
16	2-Methyl-4-phenyl-2-butanol					3.461
17	Beta-caryophyllene		0.864			
18	Jasmone					0.367
19	Allyl cyclohexylpropionate				0.660	
20	Butylated hydroxytoluene				5.348	
21	2-(Oct-2-enyl) cyclopentan-1-one					0.861
22	Methyl jasmonate					51.502
23	Methyl dihydrojasmonate					14.477
24	Alpha-hexyl cinnamaldehyde					13.328
25	Phenethyl phenylacetate					4.897
26	Germacrene D		1.834	1.424		
27	Methyl (3*Z*,7*E*,10*E*)-3,7,10,12-tridecatetraenoate		5.686			
28	1,5-Cyclooctadiene, 3-(1-Methyl-2-propen-1-yl)-		4.446			

^1^ The compounds were identified by gas chromatography–mass spectrometry (GC–MS) (QP2010 plus, Shimadzu) equipped with a DB-5 capillary column (30 m × 0.25 mm i.d., 0.25 µm film).

**Table 2:** The gastric toxicity of five Rutaceae essential oils to adult *Thrips flavus*.

Essential Oils	LC_50_ (g/L)	95% Confidence Interval	Regression Equation	Related Coefficient	*χ^2^*	*p*-Value
Orange leaf oil	0.26	0.11~0.37	*y* = 5.9807 + 1.6867*x*	0.8381	4.7591	0.1903
Zanthoxylum oil	0.27	0.16~0.36	*y* = 6.3029 + 2.3067*x*	0.8581	5.768	0.1235
Pomelo peel oil	0.44	0.35~0.51	*y* = 6.2064 + 3.3408*x*	0.9353	5.4535	0.1414
Chuan-shan pepper oil	0.58	0.51~0.69	*y* = 5.9531 + 4.0840*x*	0.9426	7.4438	0.0590
Citrus oil	2.73	1.98~3.90	*y* = 4.2585 + 1.7001*x*	0.7511	10.7618	0.0131

Note: LC_50_ = concentration to kill 50% of thrips.

**Table 3:** Control efficacy of essential oils in *Thrips flavus*.

Essential Oils	Concentration Gradients (g a.i.·hm^−2^)	Control Efficacy (%)		
		After 1 Day	After 3 Days	After 7 Days
Orange leaf oil	180.00	16.05 ± 1.24 ^b^	24.95 ± 5.40 ^c^	69.44 ± 7.35 ^c^
	360.00	38.27 ± 7.51 ^ab^	52.22 ± 5.88 ^b^	77.78 ± 3.68 ^bc^
	540.00	41.98 ± 6.88 ^ab^	56.67 ± 6.94 ^b^	93.06 ± 3.67 ^ab^
	720.00	64.20 ± 8.64 ^a^	67.78 ± 4.01 ^b^	97.22 ± 1.39 ^a^
	900.00	65.43 ± 2.47 ^a^	90.00 ± 3.85 ^a^	100.00 ^a^
Pomelo peel oil	180.00	19.75 ± 7.51 ^d^	27.40 ± 5.97 ^c^	18.37 ± 5.40 ^d^
	360.00	28.40 ± 4.45 ^cd^	54.79 ± 2.37 ^bc^	44.89 ± 1.94 ^cd^
	540.00	45.68 ± 4.94 ^bc^	76.71 ± 8.98 ^ab^	48.31 ± 2.18 ^c^
	720.00	58.02 ± 3.27 ^b^	82.19 ± 3.62 ^ab^	83.68 ± 4.08 ^b^
	900.00	90.13 ± 3.27 ^a^	95.89 ± 4.11 ^a^	100.00 ^a^
Zanthoxylum oil	180.00	15.54 ± 6.24 ^c^	34.94 ± 4.17 ^b^	48.10 ± 2.53 ^c^
	360.00	32.14 ± 2.06 ^bc^	28.92 ± 2.41 ^b^	46.84 ± 2.19 ^c^
	540.00	39.29 ± 9.45 ^bc^	54.22 ± 3.19 ^ab^	69.62 ± 4.38 ^bc^
	720.00	60.71 ± 2.06 ^ab^	69.88 ± 6.38 ^a^	87.34 ± 4.56 ^b^
	900.00	71.43 ± 5.46 ^a^	81.93 ± 9.56 ^a^	98.73 ± 1.27 ^a^
Chuan-shan pepper oil	180.00	16.67 ± 2.38 ^b^	21.69 ± 2.41 ^c^	34.18 ± 3.35 ^c^
	360.00	34.52 ± 3.15 ^ab^	43.37 ± 2.41 ^b^	51.9 ± 3.35 ^b^
	540.00	35.71 ± 4.12 ^a^	54.22 ± 4.34 ^ab^	62.02 ± 4.38 ^b^
	720.00	32.14 ± 3.57 ^ab^	48.19 ± 2.41 ^ab^	62.03 ± 2.19 ^b^
	900.00	34.52 ± 6.63 ^ab^	59.04 ± 3.19 ^a^	82.28 ± 3.35 ^a^
Citrus oil	900.00	5.95 ± 3.15 ^b^	59.04 ± 8.69 ^a^	53.16 ± 10.13 ^a^
	1800.00	26.19 ± 3.15 ^ab^	48.19 ± 6.71 ^a^	54.43 ± 5.80 ^a^
	2700.00	44.05 ± 6.30 ^a^	56.63 ± 4.17 ^a^	59.49 ± 5.06 ^a^
	3600.00	48.81 ± 7.24 ^a^	54.22 ± 6.38 ^a^	50.63 ± 9.56 ^a^
	4500.00	46.43 ± 9.45 ^a^	54.22 ± 9.64 ^a^	62.03 ± 4.38 ^a^

Note: Significant differences *(p* < 0.05) are indicated by different lowercase letters following the data in the same column for the same essential oil. The same as below.

## Data Availability

Not applicable.

## Supplementary Materials

The following supporting information can be downloaded at: https://www.mdpi.com/article/10.3390/molecules28072998/s1, Figure S1: GC–MS chromatogram of the identified chemical compounds of the five essential oils.

## Sample Availability

Samples of the compounds are available from the authors.
