# Supplementary material for: Preliminary Study on Insecticidal Potential and Chemical Composition of Five Rutaceae Essential Oils against Thrips flavus (Thysanoptera: Thripidae)
Source: Molecules. 2023 Mar 28;28(7):2998. doi: 10.3390/molecules28072998 (PMC10095842; doi:10.3390/molecules28072998)
Supplement: Supplementary file 1 [file molecules-28-02998-s001.zip › molecules-2295955-supplementary.docx]

**Figure S1 GC-MS chromatogram of the identified chemical compounds in the essential oils**

**Figure S1-1 GC-MS chromatogram of the identified chemical compounds in the citrus oil (Citrus reticulata Blanco)**

**Figure S1-2 GC-MS chromatogram of the identified chemical compounds in the pepper oil (Zanthoxylum piasezkii Maxim.)**

Note: glyceryl tricaprylate that detected at 45.052 min was added for stability by the producer.

**Figure S1-3 GC-MS chromatogram of the identified chemical compounds in the zanthoxylum oil (*Zanthoxylum bungeanum* Maxim.)**

**Figure S1-4 GC-MS chromatogram of the identified chemical compounds in the pomelo peel oil (*Citrus maxima* (Burm.) Merr.)**

**Figure S1-5 GC-MS chromatogram of the identified chemical compounds in the orange leaf oil (*Citrus sinensis* (L.) Osbeck)**
